# Marine predators segregate interspecifically by space and time in a sheltered coastal bay

**DOI:** 10.1111/jfb.15781

**Published:** 2024-05-09

**Authors:** Taryn S. Murray, Chantel Elston, Malcolm J. Smale, Amber‐Robyn Childs, Matthew L. Dicken, Paul D. Cowley

**Affiliations:** ^1^ South African Institute for Aquatic Biodiversity Makhanda South Africa; ^2^ Port Elizabeth Museum at Bayworld Gqeberha South Africa; ^3^ Department of Zoology and Institute for Coastal and Marine Research Nelson Mandela University Gqeberha South Africa; ^4^ Department of Ichthyology and Fisheries Science Rhodes University Makhanda South Africa; ^5^ KwaZulu‐Natal Sharks Board Umhlanga Rocks South Africa; ^6^ Institute for Coastal and Marine Research, Oceans Sciences Campus Nelson Mandela University Gqeberha South Africa

**Keywords:** acoustic telemetry, Acoustic Tracking Array Platform, Algoa Bay, movement ecology, social network analysis, South Africa

## Abstract

Marine predators are vital to the healthy functioning of coastal ecosystems, but to understand their roles, it is necessary to elucidate their movement ecology, particularly in relation to one another. A decade's worth of acoustic telemetry data (2011–2020) from Algoa Bay, South Africa, was investigated to determine how two mesopredatory species (teleosts: dusky kob *Argyrosomus japonicus*, *n* = 11, and leervis *Lichia amia*, *n* = 16) and two top predatory species (sharks: ragged‐tooth sharks *Carcharias taurus*, *n* = 45, and white sharks *Carcharodon carcharias*, *n* = 31) used and shared this bay ecosystem. Multi‐annual seasonal fidelity to the bay was exhibited by all species, but differences in residency were observed among species. Similarly, species used space in the bay differently—the teleosts moved less and had movements restricted to the central and western inshore regions of the bay. Conversely, the sharks roamed more, but detections were concentrated in the western part of the bay for *C*. *taurus* and in the eastern part of the bay for *C*. *carcharias*. Social network analysis showed that species segregated in space and time on a fine scale. However, there was some interaction observed between *C*. *taurus*, *L*. *amia*, and *A*. *japonicus*, but to varying degrees. This is likely because of strong habitat preferences exhibited by each species and predator–prey relationships between these predatory guilds. Results highlight that the sheltered marine Algoa Bay is a resource‐rich environment, supporting multiple predators with different hunting strategies albeit similar prey preferences. Finally, these species are likely afforded some protection by the current Greater Addo Elephant National Park Marine Protected Area in the bay but are vulnerable to fishing pressure when they leave this ecosystem.

## 1 INTRODUCTION

Marine predators can play important roles in coastal ecosystems by exerting top–down effects on prey through consumptive and non‐consumptive interactions (i.e., predation and fear‐based effects) and simulating bottom‐up processes by transferring nutrients across ecosystem boundaries (Heithaus et al., 2008; Matich et al., 2017; Papastamatiou et al., 2015). The exact nature of these roles will vary based on how predators use these ecosystems in space and time, and how they interact with each other. For example, interspecific interactions between top‐ and mesopredators can have a large influence on survival and trophic linkages, thus influencing how ecosystems function (Ritchie & Johnson, 2009).

Coastal habitats are dynamic and highly productive areas, providing a number of ecosystem services to many different species (Henseler et al., 2019; Kraufvelin et al., 2018; Lefcheck et al., 2019). Consequently, sympatric communities not only coexist but can thrive in these shared habitats (White & Potter, 2004). Despite the well‐documented occurrence of these coastal communities, our understanding of how they coexist and share resources remains limited (Heupel et al., 2019). Coastal bays, in particular, are examples of such important coastal habitats, serving as nursery, reproductive, and/or feeding grounds, with species displaying site fidelity to these defined areas. Even large coastal transient sharks, such as white *Carcharodon carcharias* and tiger *Galeocerdo cuvier* sharks, have exhibited site fidelity to specific coastal bays (Speed et al., 2010).

Although many studies have investigated movements of individual predator species in bays (e.g., Campos et al., 2009; Elston et al., 2023; Jewell et al., 2012; Kelly et al., 2007), there are only a few studies that concurrently investigate the movements of multiple predatory species in these ecosystems (Heupel et al., 2019), with no previous research on this conducted in South Africa. Some general trends indicate that species can segregate by factors such as benthos type, depth, prey availability, and physical water parameters (Speed et al., 2010). However, detailed insights into how sympatric predators may segregate or overlap in their habitat use is lacking, and this limits our understanding of ecosystem dynamics and functioning (Matich et al., 2017). Additionally, understanding movements and habitat use by multiple species is important for identifying and prioritizing areas for protection (Lea et al., 2016; Speed et al., 2010). This is particularly pertinent for marine predators, a faunal group that is, on average, threatened with extinction due to the combination of overfishing and k‐selected life histories (Dulvy et al., 2021; Essington et al., 2006).

**FIGURE 1:**
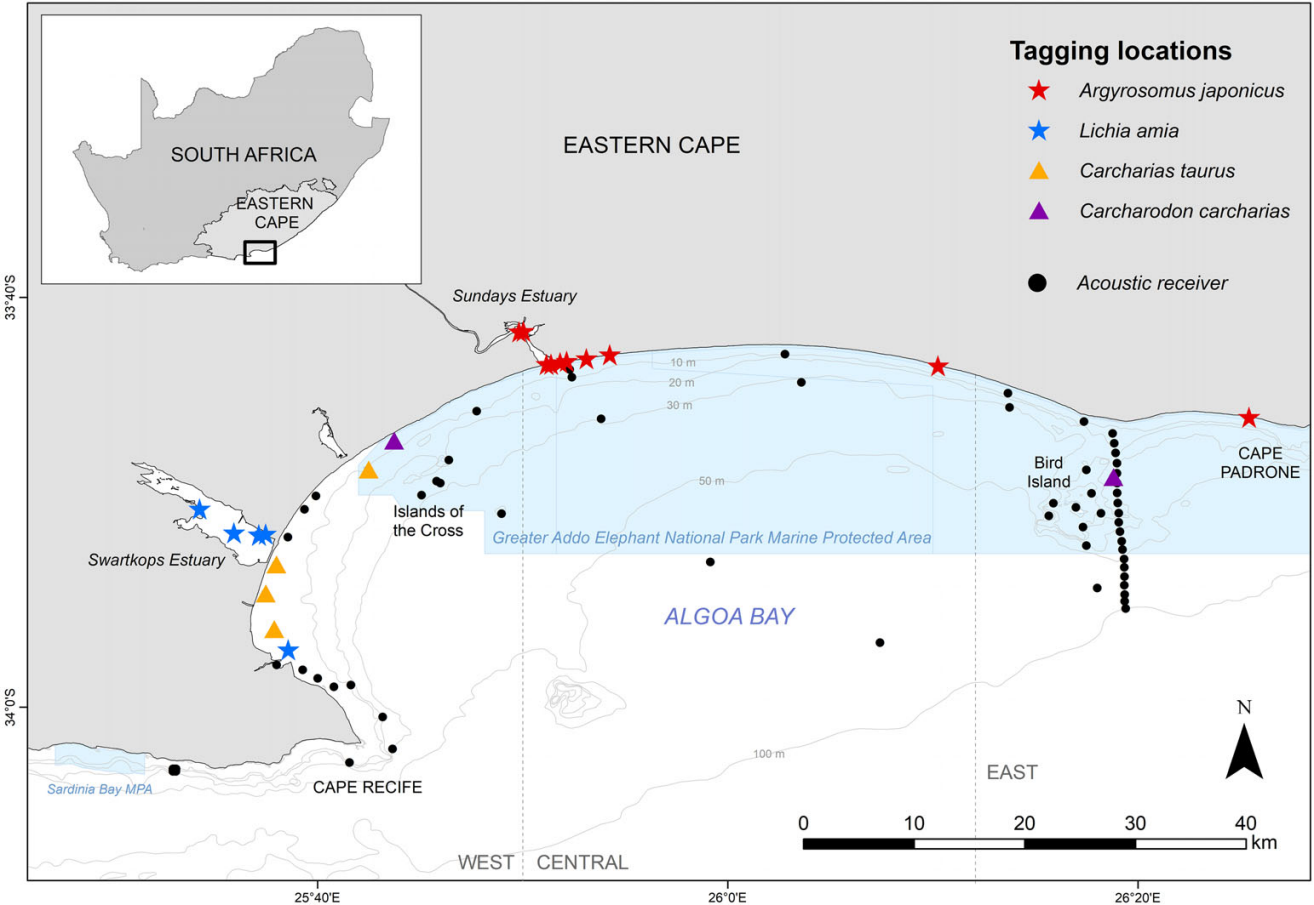
Map of Algoa Bay, South Africa, showing the location of acoustic receivers (Innovasea VR2, VR2W, and VR2AR; black dots) used to monitor the movement behavior and connectivity of tagged *Carcharodon carcharias* (purple triangle), *Carcharias taurus* (orange triangle), *Argyrosomus japonicus* (red star), and *Lichia amia* (blue star) between January 2011 and December 2020. The marine protected area (MPA) in the bay—the Greater Addo Elephant National Park MPA—is shown in light blue.

The South African coastline, spanning approximately 3000 km, is exposed to high wave energy and has few sheltered bays (Burns et al., 1999; Dames et al., 2017). On the south coast, Algoa Bay is the largest bay and functions as an ecologically important region. Consequently, it is home to numerous marine top‐ and mesopredators, including cetaceans, pinnipeds, birds, teleosts, and sharks (hosting nursery areas for the latter two) (Childs et al., 2015; Dicken, 2008; Melly et al., 2018; Murray et al., 2017; Pichegru et al., 2010; Smale et al., 2015; Stewardson, 2001). A large marine protected area (MPA)—the Greater Addo Elephant National Park (GAENP) MPA—was promulgated in 2019 (Department of Environmental Affairs, Government Notice No. 42478) in the bay (Figure 1), and the first national marine spatial plan in South Africa is currently being developed for Algoa Bay (Dorrington et al., 2018; Vermeulen‐Miltz et al., 2023). The aim of this study was to understand how predators use, and interact in, this coastal bay, using a decade's worth of acoustic telemetry data for two mesopredatory species (teleosts: dusky kob *Argyrosomus japonicus* and leervis *Lichia amia*) and two top predatory species (elasmobranchs: ragged‐tooth sharks *Carcharias taurus* and *C*. *carcharias*). Of the two teleost species under investigation, *A*. *japonicus* has been subjected to significant population declines over recent decades and their populations are collapsed (Griffiths, 1997), whereas excessive targeting effort on *L*. *amia* in recent years has raised concerns (Smith, 2008). Although *C*. *carcharias* are protected nationally (since 1991), a recent decline in their numbers along the South African coastline is a cause for concern (Bowlby et al., 2023), and *C*. *taurus* are heavily targeted by the competitive recreational fishing fraternity, and fall victim to the bather protection nets along the east coast during their reproductive migrations (Dudley & Simpfendorfer, 2006). Results from previous studies using dart tagging and acoustic telemetry methods suggest that adult *A*. *japonicus* and *C*. *taurus* may remain resident to Algoa Bay throughout the year (Childs, 2013; Childs et al., 2015; Smale et al., 2015). *C*. *taurus* and *C*. *carcharias* display seasonal changes in abundance (Dicken et al., 2013; Smale et al., 2015), and *L*. *amia* undertake annual spawning migrations to KwaZulu‐Natal (KZN, Dunlop et al., 2015), returning in the austral summer. However, limited information is available on how various life stages of these four species simultaneously utilize Algoa Bay seasonally and over multiple years. As such, their movements and space use relative to one another were quantified. The specific aims were to assess the predators' seasonal presence to Algoa Bay, identify their high use areas, quantify their connectivity patterns and spatial extent of their movements in the bay, and determine whether predators segregated in space and time. Additionally, given the relatively recent promulgation of the GAENP MPA, gaining an understanding of the movements of these species in Algoa Bay is critical to understanding the relative efficacy of the MPA.

## 2 MATERIALS AND METHODS

### 2.1 Study site

Algoa Bay is the largest bay along the southern coast of South Africa. It faces a southeast direction, is relatively shallow with depths not exceeding 80 m, and measures approximately 75 km between the low‐lying Cape Recife on the western side and the less prominent, slightly elevated Cape Padrone on the eastern side (Figure 1) (Goschen et al., 2012; Roberts, 2010). The water temperature fluctuates seasonally, with temperature structures being far more intense in summer due to the prevalence of easterly winds (Goschen et al., 2012). Five estuaries occur in Algoa Bay; however, only the predominantly open Swartkops and Sundays estuaries are considered functional, providing major freshwater output into the bay (Nodo, Childs, Pattrick, & James, 2023). Both systems are heavily impacted by anthropogenic activities, either being agriculturally influenced (Sundays) or urbanized and heavily polluted (Swartkops), and experience continuous eutrophic conditions, resulting in phytoplankton blooms and associated depletion of bottom‐water oxygen (Nodo, Childs, Pattrick, Lemley, & James, 2023). Two island groups are located in Algoa Bay; the Island of the Cross on the western side, consisting of three small outcrops, namely St Croix, Jahleel, and Brenton islands, and the Bird Island group on the eastern side, comprising Bird, Stag, and Seal islands (Figure 1). Almost 40% of the bay is currently protected by the GAENP MPA (Figure 1).

### 2.2 Acoustic array and animal tagging

An array of 59 acoustic receivers (Innovasea VR2, VR2W, and VR2AR), deployed since 2008 as part of ongoing research, was used to monitor the movements of tagged *A*. *japonicus*, *L*. *amia*, *C*. *taurus*, and *C*. *carcharias* in Algoa Bay for a period of 10 years (2011–2020) (Figure 1). Receivers were downloaded every 6–12 months. Due to the dynamic nature of the marine environment, some receivers were lost and/or malfunctioned in which case the data from these receivers were omitted from the dataset.

Ethical approval for this study was received from the South African Institute for Aquatic Biodiversity's Animal Ethics Committee (#2010/02, #2012/12 and #2013/06). Bayworld ethics committee approved shark sampling under the SOP for shark tagging. Sampling and tagging were conducted under Department of Forestry, Fisheries and the Environment permits (RES 2009/42, RES 2010/38, RES2011/33, RES2012/09, RES2013/01, RES 2013/19, RES2014/15, RES2014/23, RES 2015/34, and RES 2015/55).

Subadult and adult *A*. *japonicus* and *L*. *amia* (Table 1) were caught from the shore (marine coastline) or boats (in estuaries) using conventional fishing tackle and were surgically implanted with acoustic transmitters following the procedure outlined by Cowley et al. (2008). Briefly, fish were placed in a tank containing estuarine or seawater (dependent on the site of capture) and anaesthetized using 2‐phenoxyethanol (approximately 0.5 mL · l^−1^). Fish were measured to the nearest millimeter (total length [TL] for *A*. *japonicus* and fork length [FL] for *L*. *amia*) and placed ventral side up on high‐density V‐shaped foam. A small incision (1.5–2 cm) was made on the ventral surface of the fish behind the pelvic girdle, a transmitter (Innovasea V13‐1 L, V16‐4 L, V16‐4H, see Table S1 in the supplement) was inserted into the body cavity, and the incision was closed using two independent CliniSilk sutures after which an antiseptic wound powder, which turns to a gel upon wetting, was placed over the closed incision. After surgery, the fish was placed in a recovery bath filled with either fresh estuarine or seawater, and once recovered, it was released into the estuary or sea at the capture site.

**TABLE 1:** Numbers and details of four predatory species (*Argyrosomus japonicus*, *Lichia amia*, *Carcharias taurus*, and *Carcharodon carcharias*) tagged and monitored using acoustic telemetry in Algoa Bay, South Africa, between 2011 and 2020.

Species	Life stage	No. tagged	No. (%) detected and included in analyses
*Argyrosomus japonicus*	Subadult (70–90 cm TL)	8	2 (25)
	Adult (>90 cm TL)	14	9 (64)
*Lichia amia*	Subadult (40–70 cm FL)	13	7 (54)
	Adult (>70 cm FL)	10	9 (90)
*Carcharias taurus*	Juvenile (<180 cm TL)	6	6 (100)
	Subadult male: 180–220 cm TL	10	10 (100)
	Subadult (female: 180–240 cm TL	17	16 (94)
	Adult male: >220 cm TL	0	0
	Adult female: >240 cm TL	6	6 (100)
*Carcharodon carcharias*	Juvenile (<300 cm TL)	27	23 (85)
	Subadult male: 300–360 cm TL	3	3 (100)
	Subadult female: 300–480 cm TL	17	14 (82)
	Adult male: >360 cm TL	0	0
	Adult female: >480 cm TL	1	1 (100)

Abbreviations: FL, fork length; TL, total length.

Juvenile, subadult, and adult *C*. *taurus* and *C*. *carcharias* (Table 1) were captured offshore from boats using fishing rods or handlines and barbless “J” hooks baited with fish, or drum lines with barbless circle hooks. All *C*. *taurus* and four *C*. *carcharias* had acoustic transmitters (Innovasea V16‐5H, V16‐6 L, see Table S1 in the supplement) surgically implanted following the procedure outlined by Smale et al. (2015). After capture, sharks were rapidly brought to the surface, swum into a PVC hoop net, and brought aboard the boat. Alternatively, they were brought alongside a boat and held in a partially submerged cradle to reduce stress and facilitate surgery. They were immediately inverted to induce tonic immobility, measured to the nearest centimeter TL. The hook was removed from the mouth and a 2.5‐cm incision was made in the abdominal wall to allow for tag insertion. The wound was sealed with three CliniVet monofilament sutures. Sharks were returned to the water after approximately 10 min. The remaining *C*. *carcharias* (*n* = 33) were tagged externally following Kock et al. (2013). The sharks were attracted to a boat in offshore waters using bait and chum, and the length was estimated to the nearest 0.5 m TL (as in Kock et al., 2013). A modified spear gun was used to deploy the acoustic transmitter (Innovasea V16‐5H, V16‐6 L, see Table S1 in the supplement), which was encased in the manufacturer's “shark case” for added protection, into the musculature at the base of the first dorsal fin.

### 2.3 Data analyses

To address marine use in Algoa Bay, only marine detections were considered (estuary detections for *A*. *japonicus* and *L*. *amia* were removed from analyses). Individuals that were detected for fewer than 10 days on marine receivers were removed from analyses to reduce the bias introduced by having a very small number of detections and to prevent spurious results. All analyses, including model assumptions and diagnostic plots, were conducted in R (R Core Team, 2023) unless otherwise stated. If statistical assumptions of parametric tests were violated, equivalent non‐parametric statistics were employed.

#### 2.3.1 Residency to Algoa Bay

Detection indices (DIs) were calculated in two ways (Appert et al., 2023) for individuals to determine how often they were detected in Algoa Bay. First, the DI_total_ was defined as the number of days an individual was detected on a receiver in Algoa Bay as a proportion of the total number of days monitored (i.e., number of days from tagging date to the end of the study period, or to the date of recapture, or to the date of tag battery expiration, or the known date of last detection on a receiver outside of Algoa Bay), a more conservation approach. Second, to assess the relative residency while in the bay, DI_bay_ was calculated as the number of detected days in Algoa Bay divided by the number of days at liberty until last detection in Algoa Bay. To determine if the mean DI_bay_ between species and between life stages was significantly different, non‐parametric Mann–Whitney *U* tests or Kruskal–Wallis test by ranks followed by the Dunn test were performed (with *p*‐values adjusted with the Benjamini–Hochberg method to reduce the chance of type 1 errors, i.e., false positives). Daily detection plots were used to graphically represent temporal use of the bay and were spatially represented in terms of western, central, and eastern regions of the bay (see Figure 1).

Monthly DIs were calculated for individuals, defined as the proportion of days detected while in Algoa Bay in a given month. To determine if species displayed seasonal trends in presence in the bay, a Generalized Additive Mixed Model (GAMM) using the R package “mgcv” (Wood, 2011) was used to test if monthly DIs significantly differed between species and month. The number of days detected per individual in each month was chosen as the response variable, and an offset of the total number of days in each month was included to take into account that these differ. The response variable was an overdispersed count variable and as such a quasipoisson distribution was selected. Individual was added as a random effect in the model. The GAMM was laid out as follows:

$$\begin{array}{l} \mathrm{No}.\;\text{of days detected in month}\sim \text{species}\\ \;+\;s\left(\text{month,}k=12,\mathrm{by}=\text{species,}\mathrm{bs}=\mathrm{cc}\right)+s\left(\text{individuals,}\mathrm{bs}=\mathrm{re}\right)\\ \;+\;\text{offset}\left(\log\left(\mathrm{no}.\;\text{of days in each month}\right)\right),\;\text{family}=\text{quasipoisson} \end{array}$$

#### 2.3.2 Spatial use of Algoa Bay

A permutational MANOVA (PERMANOVA) (Anderson, 2001) was used to determine if different species and life stages used significantly different areas of the bay. The proportion of detections at each receiver for each individual was calculated and was used to develop a Euclidean dissimilarity matrix. The PERMANOVA used the dissimilarity matrix to test whether spatial receiver use significantly differed between species, life stages, and tagging regions (i.e., whether tagged in an estuary or in the coastal region of the bay), and the interactions between these factors, using the R package “vegan” (Anderson, 2001).

A network approach was then adopted to visualize and quantify connectivity and space use in the bay (Lédée et al., 2015). Networks were constructed whereby receivers were considered as nodes, and movements (and therefore detections) between two receivers by an individual in a 24‐h period were considered as edges in the networks. Networks were aggregated by species and life stages to visualize space use between these groups, but individual networks were constructed to quantify space use. Two network metrics were calculated for individual networks to determine the extent of space use in the bay: (1) node density, which is the proportion of available nodes in the network that were used and (2) edge density, the proportion of available edges in the network used. Networks and metrics were constructed using the R package “igraph” (Csardi & Nepusz, 2006). Mann–Whitney *U* tests or Kruskal–Wallis test by ranks with post hoc Dunn's test were used to determine if network metrics were significantly different between species and life stages.

#### 2.3.3 Segregation in space and time

A social network analysis approach was used to determine whether species and life stages segregated spatially and temporally on a fine scale. In this approach, individuals were treated as nodes, and edges were defined when individuals were detected at the same receiver within an hour time window. One year's worth of telemetry data, collected from October 1, 2014, to September 30, 2015, were used to construct the network, as this was when most individuals were tagged and were actively being detected in the bay. Networks were constructed using the R package “asnipe” (Farine, 2013), and edges in the network were weighted by the simple‐ratio association index that ranges from 0 (two individuals never co‐occurred in the same group) to 1 (individuals always co‐occurred in the same group). The assortativity coefficient, based on weighted edges, was then calculated using the R package “assortnet” (Farine, 2014). This index is a measure of the tendency of nodes in a network to be connected to other nodes that have the same/similar movement or connectivity traits (which can be categorical or continuous). The assortativity coefficients were calculated for the traits of species and life stages, and their significance was tested using a node permutation method. This consisted of randomly reallocating the phenotype of the nodes while maintaining the same edge structure of the network. The randomization was repeated 1000 times, and a *p*‐value was obtained by comparing the randomly generated coefficient values to the observed ones (Farine & Whitehead, 2015).

## 3 RESULTS

### 3.1 General

**FIGURE 2:**
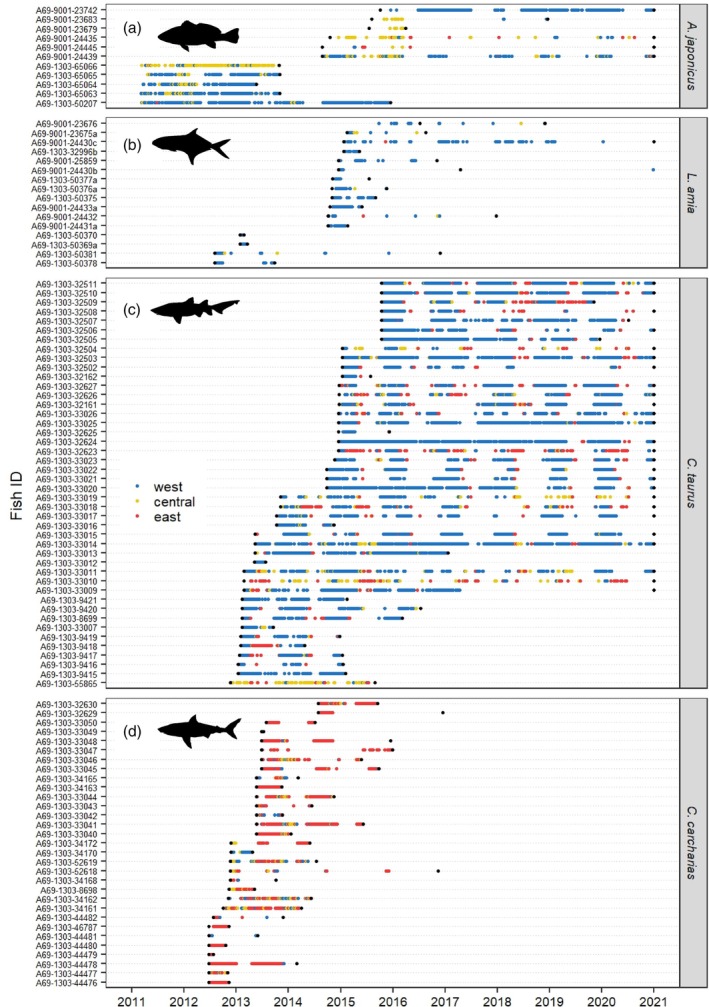
Daily detection plot for the four predatory species (a) *Argyrosomus japonicus*, (b) *Lichia amia*, (c) *Carcharhias taurus*, and (d) *Carcharodon carcharias* tagged and detected using acoustic telemetry in Algoa Bay, South Africa, for a period of 10 years (2011–2020). Colors represent the part of the bay in which receivers were deployed, namely west (blue), central (yellow), and east (red). Black circles on either side of the detections indicate date of tagging (left circle) and battery expiration, recapture, date of last detection outside Algoa Bay, or end of the study period (if transmitters were still active, right circle).

From January 2010 to December 2020, 133 mesopredatory teleost fish and top predatory sharks were tagged and monitored in Algoa Bay (Table S1), although only data from 103 (77%) individuals were included in analyses given the lack of detections from some individuals (Table 1; Figure 2).

Twelve teleosts were recaptured prior to transmitter battery depletion. These included five (20%) *A*. *japonicus* that were recaptured, on average (±SD), 60 days (±18 days) after release, and seven (30%) *L*. *amia* that were recaptured, on average (±SD), 190 days (±184 days) after release (see Table S1 in the Supplement). One *C*. *carcharias* died approximately 2 years after being tagged and washed up ashore in Algoa Bay in June 2015, and all externally tagged *C*. *carcharias* were suspected to have lost their transmitters approximately 375 days (±249 days) after tagging despite most having relatively long (2.5–4.2 years) expected battery lives.

### 3.2 Residency to Algoa Bay

**FIGURE 3:**
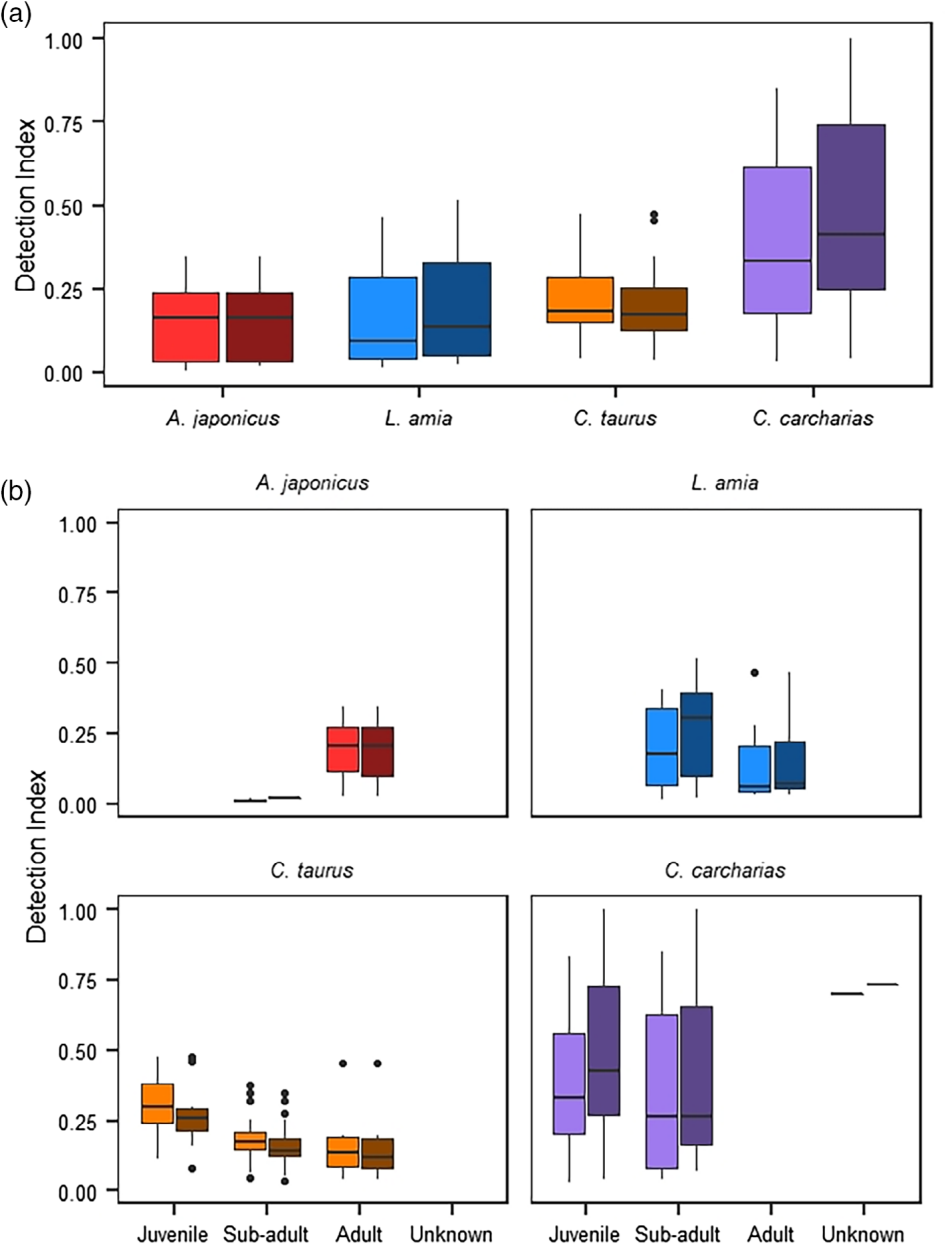
Boxplots showing (a) both the overall detection indices (lighter shades) and the detection indices when in the bay (darker shades), as well as (b) by life stage (juvenile, subadult, adult, unknown) for the four predatory species (*Argyrosomus japonicus* [red], *Lichia amia* [blue], *Carcharias taurus* [orange], and *Carcharodon carcharias* [purple]) detected in Algoa Bay, South Africa, between 2011 and 2020.

All species were monitored for extended periods of times (mean days at liberty for each species ranged from 613 (*L*. *amia*) to 1687 (*C*. *taurus*) days), but overall rates of detection to the bay were generally low to moderate, with species being detected, on average, for ~15%–39% of the days they were monitored. Overall DIs (DI_total_) to the bay varied among species (Figure 3a), with *C*. *carcharias* being detected most frequently (mean DI_total_: 0.392, range: 0.032–0.852) followed by *C*. *taurus* (mean DI_total_: 0.216, range: 0.041–0.474), *L*. *amia* (mean: 0.166, range: 0.014–0.467), and *A*. *japonicus* (mean: 0.152, range: 0.006–0.347). Although there was a significant difference in DI_total_ among species (Kruskal–Wallis *χ* ^2^ = 22.08, *df* = 3, *p* < 0.001), the post hoc Dunn's test revealed that only *C*. *carcharias* and all other species (*A*. *japonicus*: *z* = −3.458, *p* = 0.001; *L*. *amia*: *z* = 3.346, *p* = 0.002; *C*. *taurus*: *z* = 3.958, *p* < 0.001) were significantly different. DI_bay_ followed a similar pattern to DI_total_ for all species (Figure 3a), with *C*. *carcharias* being detected most frequently (mean DI_bay_: 0.472, range: 0.042–1.000) and *A*. *japonicus* the least frequently detected (mean DI_bay_: 0.152, range: 0.019–0.347). Similar to DI_total_, there was a significant difference in DI_bay_ among species (Kruskal–Wallis *χ* ^2^ = 14.787, df = 3, *p* = 0.002), and the post hoc Dunn's test revealed that only *C*. *carcharias* and all other species (*A*. *japonicus*: *z* = −2.923, *p* = 0.010; *L*. *amia*: *z* = 3.205, *p* = 0.008; *C*. *taurus*: *z* = 2.441, *p* = 0.029) were significantly different.

Both DI_total_ and DI_bay_ between life stages were significantly different for *C*. *taurus* only (DI_total_: Kruskal–Wallis *χ* ^2^ = 9.605, *df* = 2, *p* = 0.008; DI_bay_: Kruskal–Wallis *χ* ^2^ = 9.137, *df* = 2, *p* = 0.010), with juvenile *C*. *taurus* being detected more frequently, on average, compared to subadults (DI_total_: *z* = 2.897, *p* = 0.011; DI_bay_: *z* = 2.828, *p* = 0.014) and adults (DI_total_: *z* = −2.308, *p* = 0.031; DI_bay_: *z* = −2.247, *p* = 0.037) (Figure 3b). *A. japonicus* was excluded from life‐stage comparisons because of a relative lack of subadults detected on marine receivers.

**FIGURE 4:**
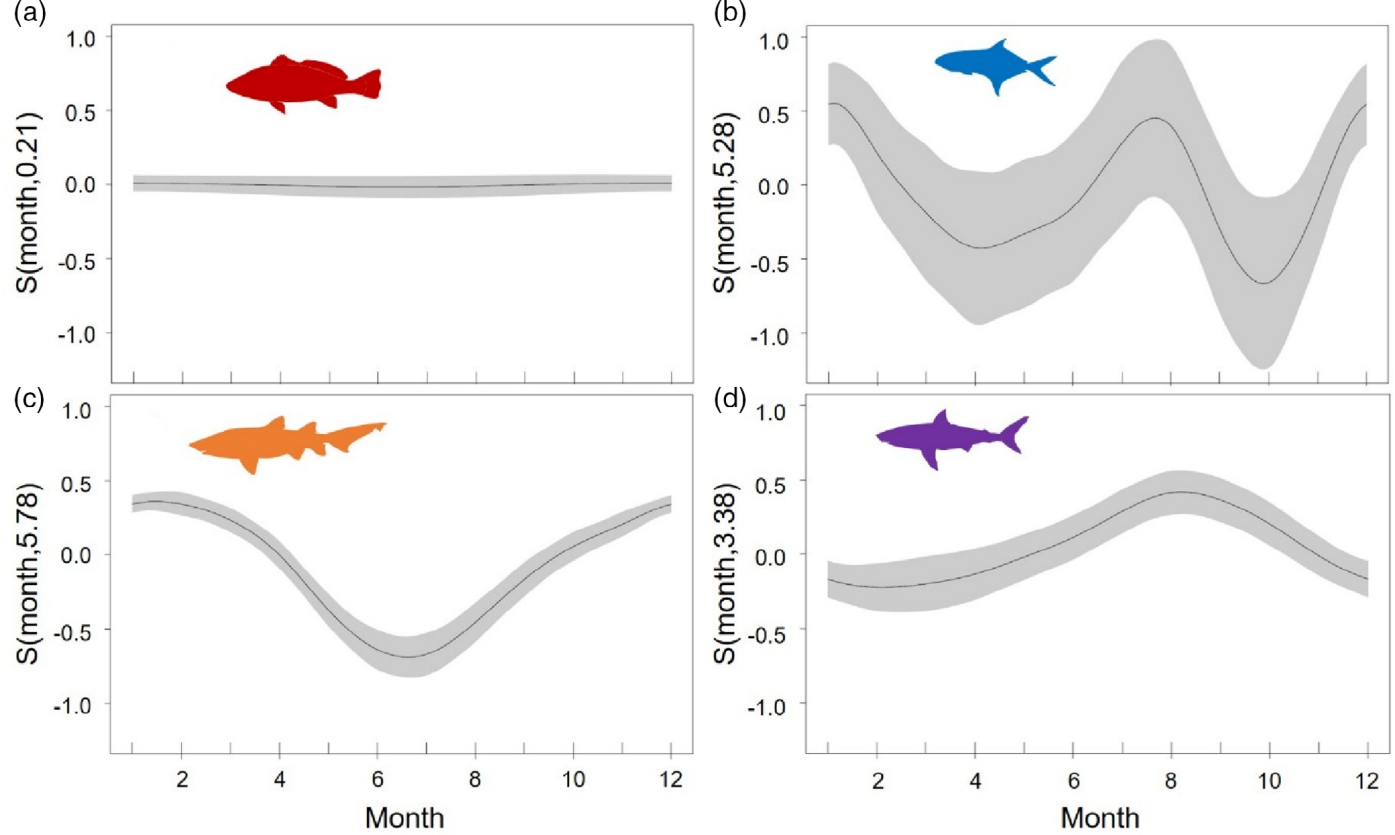
The probability of detection of the four predatory species in Algoa Bay, South Africa, by month, as predicted by the generalized additive mixed model. (a) = *Argyrosomus japonicus*, (b) = *Lichia amia*, (c) = *Carcharias taurus*, (d) = *Carcharodon carcharias*.

Even though there was variation in the presence of each species and life stage in the bay, all species, except *A*. *japonicus* (Figure 4a), displayed a significant seasonal, yet variable, peak in the probability of being detected in the bay (Table 2; Figure 4). *C. taurus* were most often detected in summer (Figure 4c), *C*. *carcharias* were most often detected in late winter/spring (Figure 4d), and *L*. *amia* were most often detected in winter and summer (Figure 4b).

**TABLE 2:** Output of the generalized additive mixed model investigating whether *Argyrosomus japonicus*, *Lichia amia*, *Carcharias taurus*, and *Carcharodon carcharias* displayed seasonal trends in presence to Algoa Bay, South Africa, between 2011 and 2020.

No. days detected ~ species + s(month, *k* = 12, by = species, bc = “cc”) + s(individual, bs = “re”) + offset(log[no. days in month])			
Term	EDF	*F*	*p*‐Value
s(month): *Argyrosomus japonicus*	0.2	0.025	0.32
s(month): *Lichia amia*	445.3	2.81	<0.01*
s(month): *Carcharias taurus*	5.8	42.20	<0.01*
s(month): *Carcharodon carcharias*	3.4	5.70	<0.01*
Adjusted *R* ^2^	0.36		
Deviance explained	39.4%		

*Note*: Asterisks (*) denote significant *p*‐values.

Abbreviations: EDF, Effective degrees of freedom.

### 3.3 Spatial use of Algoa Bay

**FIGURE 5:**
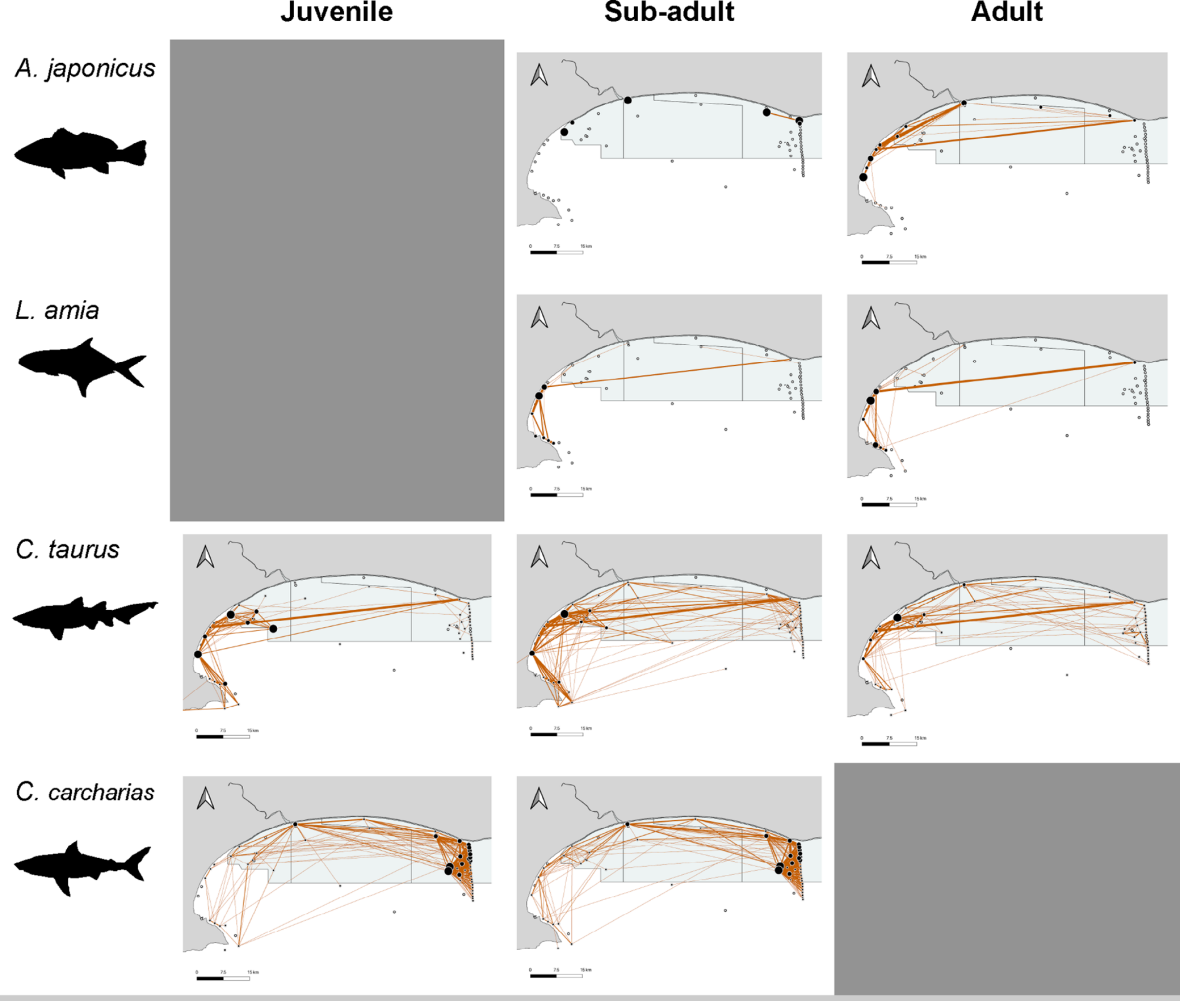
Movement networks aggregated by species and life stages (juvenile, subadult, adult) for four predatory species (*Argyrosomus japonicus*, *Lichia amia*, *Carcharias taurus*, and *Carcharodon carcharias*) in Algoa Bay, South Africa, between 2011 and 2020. Connections (edges) between receivers (nodes) indicate subsequent detections that occurred within 24 h. The width of edges represents the number of times an edge occurred. The size of the nodes denotes the proportions of detections at that node.

Both teleost species used less space compared to the sharks, being detected on different receivers in significantly different proportions (Table 3), and detections were restricted to the shallow inshore receivers in the central (*A*. *japonicus*) and western (*L*. *amia*) parts of the bay (with a few detections in the eastern inshore region) in regions where they were tagged. *C. taurus* were largely recorded on receivers positioned in the western part of the bay, but unlike *L*. *amia*, they were extensively recorded on receivers located further offshore. *C. carcharias* were recorded predominantly in the eastern part of the bay (Figure 5).

**TABLE 3:** Permutational MANOVA (PERMANOVA) results on the proportions of detections for each individual at each receiver in Algoa Bay, compared between species (*Argyrosomus japonicus*, *Lichia amia*, *Carcharias taurus*, and *Carcharodon carcharias*) and life stages (juvenile, subadult, and adult).

Term	*df*	F‐model	*R* ^2^	*p*‐Value
Species	3	23.65	0.41	0.001*
Life stage	2	2.53	0.03	0.004*
Tag region	1	1.85	0.01	0.093
Species × life stage	3	2.61	0.04	0.001*
Species × tag region	1	1.47	0.01	0.155
Residuals	84	0.21	0.49	
Total	94	35.33	1	

*Note*: Asterisks (*) denote significant *p*‐values.

**FIGURE 6:**
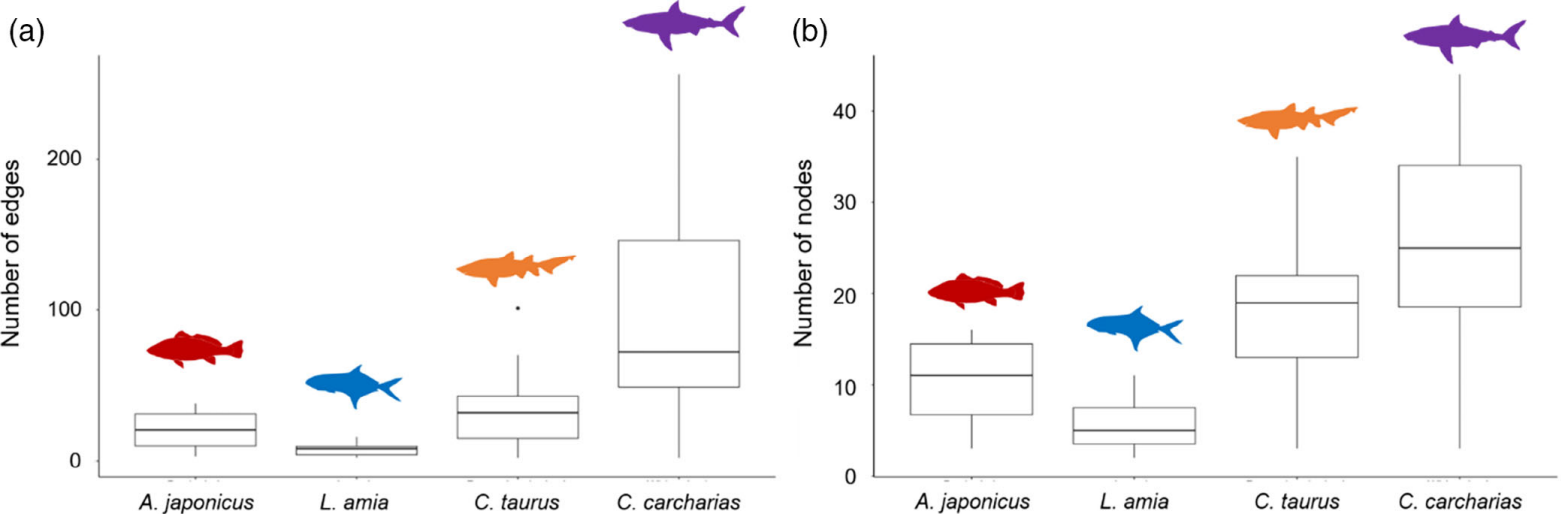
The number of edges (a) and nodes (b) used by individuals in the movement network analyses, grouped by species (*Argyrosomus japonicus*, *Lichia amia*, *Carcharias taurus*, and *Carcharodon carcharias*) to illustrate connectivity among acoustic receivers deployed in Algoa Bay, South Africa, between 2011 and 2020.

The sharks, in general, exhibited much higher levels of connectivity compared to the teleosts. Species were also different in the extent of space used, with edge (Kruskal–Wallis *χ* ^2^ = 46.98, *df* = 3, *p* < 0.01) and node (Kruskal–Wallis *χ* ^2^ = 41.107, *df* = 3, *p* < 0.01) densities from individual networks being significantly different across most species comparisons. The space used by *C*. *carcharias* was significantly different compared to *A*. *japonicus* (edge, node: *z* = −2.92, −2.91; *p* < 0.01, < 0.01), *L*. *amia* (edge, node: *z* = −6.61, −6.19; *p* < 0.01, < 0.01), and *C*. *taurus* (edge, node: *z* = −4.22, −2.87; *p* < 0.01, < 0.01). *L. amia* displayed the lowest edge and node densities, with *C*. *carcharias* displaying the highest (Figure 6a,b). Levels of connectivity exhibited among life stages differed between species (Figure 5), but this was not significant and was not reflected in the extent of space used.

### 3.4 Segregation in space and time

**FIGURE 7:**
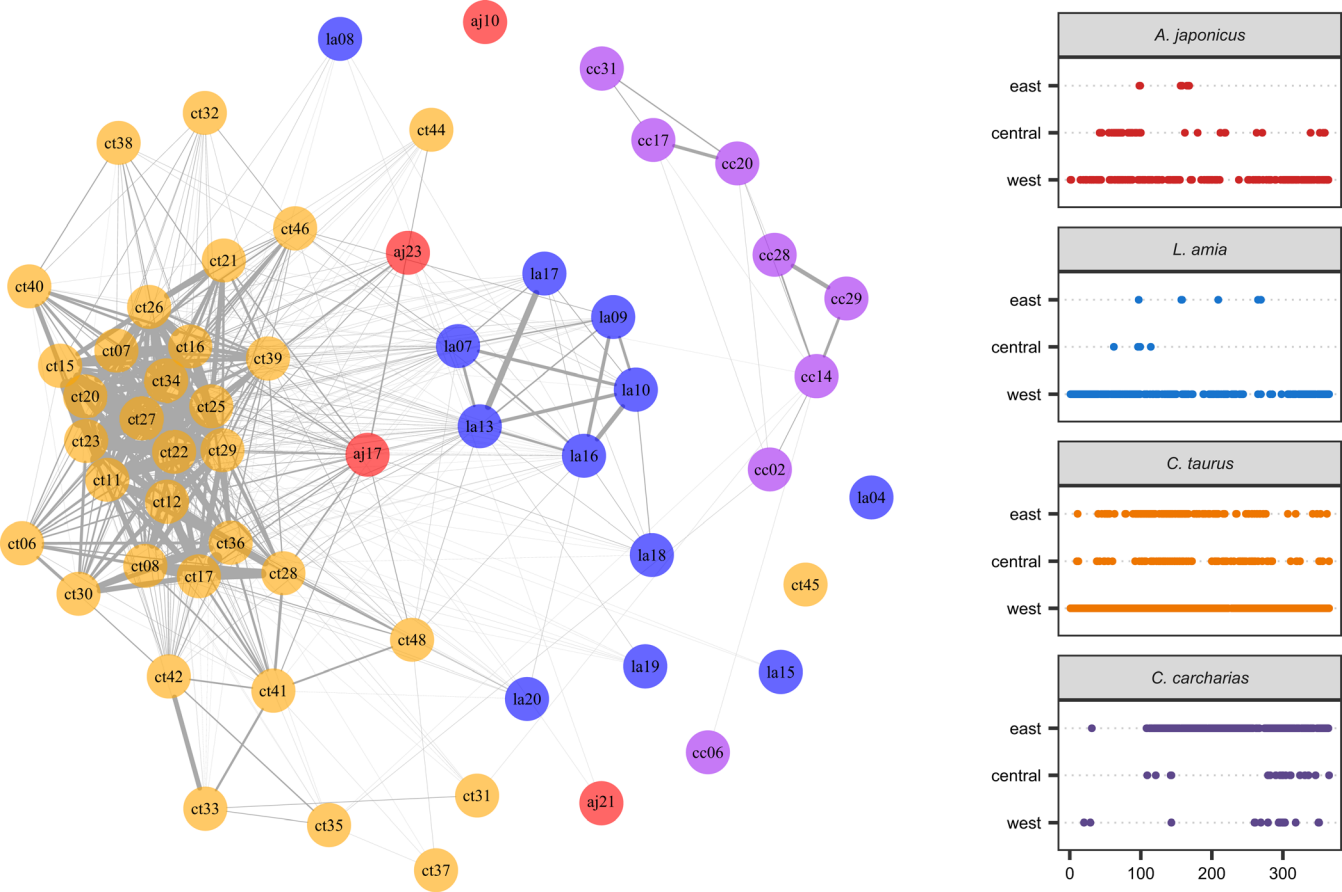
Social network analysis for four predatory species detected in Algoa Bay, South Africa, for a period of 1 year (October 1, 2014–September 30, 2015). Connections between nodes/individuals represent when individuals co‐occurred at the same acoustic receiver in the space of 1 h. Colors represent the different species: aj = *Argyrosomus japonicus* (red), la = *Lichia amia* (blue), ct = *Carcharias taurus* (orange), cc = *Carcharodon carcharias* (purple). Additionally, a daily detection plot for all individuals per species detected in each region of the bay is presented on the right.

Four *A*. *japonicus*, 12 *L*. *amia*, 34 *C*. *taurus*, and 8 *C*. *carcharias* were detected between October 1, 2014, and September 30, 2015, and were included in the social network analysis. Individuals were found to significantly co‐occur more frequently in space and time with conspecifics over heterospecifics (assortativity coefficient = 0.323, *p* < 0.01, Figure 7). For example, individual *C*. *taurus* used similar areas of the bay (with little variability among individuals) relative to the other species, being recorded predominantly on the same receivers positioned on the western side of the bay. Co‐occurrence among *A*. *japonicus* individuals was considerably lower (Figure 7). Conversely, there was no significant association between individuals of the same life stages (assortativity co‐efficient = −0.019, *p* = 0.14).

## 4 DISCUSSION

Understanding fish movement ecology is critical for ecosystem functioning and species persistence (Cooke et al., 2022). It also requires a broad context, something that is difficult to achieve in single‐species research. This study used a decade's worth of telemetry data to show that four sympatric predators, two top‐ and two mesopredators, segregated in both space and time in a large South African coastal bay. This segregation is likely driven by each species' unique ecological requirements and predator–prey dynamics and highlights that Algoa Bay is a resource‐rich environment, supporting different predatory guilds. These insights are valuable in refining the roles of these different predators and the broader functioning of this bay ecosystem.

All four predatory species were monitored across multiple years in Algoa Bay, and most of them were detected at relatively equal rates. The exception was between *L*. *amia* and *C*. *taurus*, with the latter being detected far more frequently (over longer periods of time). Species were detected in the bay, on average, between 15% and 39% of the days they were monitored. Although it is difficult to determine true levels of residency to the bay given the relatively sparse nature of the receiver network, it is likely that all of these species were absent from the marine coastal environment of Algoa Bay for certain periods of time. In particular, both juveniles and adults of the mesopredatory teleost species, *A*. *japonicus* and *L*. *amia*, are known to frequently use estuaries in addition to the coastal surf zone (Childs et al., 2015; Murray et al., 2017). Indeed, although estuary detections were not considered in this study (given the focus was interspecific interactions in the marine environment, and both shark species do not use estuaries [*C*. *taurus* to a small degree]), many tagged individuals were detected in the estuaries of Algoa Bay (ATAP, unpublished data). Similarly, both shark species are known to undertake annual migrations to other areas along the coast. *C. taurus* are known to migrate toward the east coast (Smale et al., 2015), and the movement of *C*. *carcharias* is characterized by periods of fidelity at aggregation sites interspersed by periods of sustained swimming (Bonfil et al., 2005; Bruce et al., 2006; Weng et al., 2007). Consequently, given the reliance on other habitats by all four species, the relatively low and equal detection rates were unsurprising. However, most individuals were detected in Algoa Bay for multiple months or years, showing it is an important site for these species to which they display site fidelity.

Distinct seasonal trends in detections and space use were observed within and among. *C*. *taurus* were most often detected in summer, with *C*. *carcharias* in late winter/early spring. *L. amia* had two detection peaks in summer and winter, which could reflect the beginning and end of their known annual migration, moving from the south coast (where Algoa Bay is) to the east coast of South Africa in winter (Mxo, 2023; van der Elst et al., 1993).

*L. amia* and *C*. *taurus* had detection peaks in summer, and both species had large portions of their detections in the western part of the bay. It is likely that oceanographic conditions of the bay contributed to these observed seasonal peaks and spatial distribution. During summer, easterly winds dominate in and around Algoa Bay, resulting in the development of wind‐driven upwelling cells and much colder waters in the eastern part of the bay (Elston et al., 2023; Goschen et al., 2012). Therefore, the tagged individuals may be behaviorally thermoregulating, as seen in many ectothermic species (Matern et al., 2000; Speed et al., 2012), and they could potentially be restricting their movements to the warmer inner western part of the bay, where receivers are abundant, resulting in higher detection rates in summer. Unlike the thermoregulatory *C*. *carcharias* (Goldman, 1997), who mostly used the eastern part of the bay, it is possible that *C*. *taurus* and *L*. *amia*, who do not have the ability to thermoregulate, used the western part of the bay in summer as a thermal refuge. Apart from oceanographic factors, it appears that biological factors and habitat requirement played a role in the temporal and spatial use of the bay by these four species. For example, Algoa Bay is thought to be a nursery area for *C*. *taurus*, where pregnant females are presumed to pup in summer (Smale et al., 2015). This would corroborate the peak in summer detections seen in this study. However, this is difficult to confirm, as only a small number of adult females *C*. *taurus* were tagged and monitored for extended periods of time. The increased presence of *C*. *taurus* in the western portion of the bay may also be related to fidelity to specific reefs as this region has a high number of reefs with receivers situated close to these reefs (ATAP, personal communication). This species is also perceived to be docile and sluggish (Bass, 1978; Fox et al., 2009), resting during the day around reefs (Smale et al., 2015). Space use within the bay by the two teleost species may have been attributed to habitat requirements and, hence, tagging locations.

The vast majority of *A*. *japonicus* were tagged in around the Sundays Estuary in the central region of the bay, and most *L*. *amia* were tagged in and around the Swartkops Estuary in the western part of the bay. As these species display fidelity to specific estuaries (Childs et al., 2015; Murray et al., 2017), it is likely that they moved in and out of their tagging estuaries, subsequently being frequently detected on receivers deployed in the nearby marine environment. Location and depths of the deployed receivers and spatial constraints of the receiver array may have also influenced the differing space use of the bay by sharks and teleosts. Both *L*. *amia* and *A*. *japonicus* swim close to the backline of the surf zone, often out of detection range of receivers deployed (which were all in depths of 10 m or more), although both sharks used significantly more space and were frequently detected on both inshore and offshore receivers in the bay.

Prey availability is also known to be a major driver of spatial requirement in marine predators (Gallagher et al., 2022). It is thus likely that the observed peak in late winter/early spring in the eastern part of the bay by *C*. *carcharias* was driven by prey availability. This part of the bay is home to Bird Island and South Africa's most easterly population of Cape fur seals *Arctocephalus pusillis* (Kirkman et al., 2012). In winter, the seal pups begin to enter the water after weaning (Kirkman et al., 2006) and provide easier foraging opportunities compared to their adult counterparts (Le Boeuf & Crocker, 1996). Previous studies in other parts of South Africa have identified changes in seasonal presence for *C*. *carcharias* in association with seal colonies (Kock et al., 2013; Laroche et al., 2008; Towner et al., 2016). It is thus plausible that *C*. *carcharias* are aggregating in the eastern part of Algoa Bay in winter/early spring to prey on YOY seals.

From a fine‐scale perspective, interspecific segregation in space and time was also observed by tagged species, despite evidence of large‐scale overlap in habitat use among species (e.g., *L*. *amia* and *C*. *taurus* having most of their detections in the western portion of the bay). In particular, over the course of 1 year, species were found to associate significantly more frequently with conspecifics over heterospecifics. All these species adopt relatively different hunting strategies but have a large overlap in preferred prey items, so they may be segregating in habitat. For example, *C*. *taurus* were recorded predominantly on receivers deployed in reef habitat compared to the teleosts, which were recorded mostly on receivers deployed in inshore habitats (ATAP, personal communication), but all have been recorded eating smaller fish species among other things (Smale, 2005). This is unusual as most other studies have shown sympatric predators to partition prey but have overlapping habitat use or vice versa. For example, juvenile sharks in Moorea, French Polynesia (Matich et al., 2017) shared habitat but consumed different prey, whereas reef sharks in the southern Great Barrier Reef, Australia, segregated by habitat use but consumed similar prey (Heupel et al., 2018). This likely reflects a strong habitat preference exhibited by each tagged species in this study, with different habitats in the bay providing the necessary resources and optimal conditions for each species at different times. This variability in resource use likely promotes the coexistence of multiple predatory species in the bay.

It is likely that the observed fine‐scale segregation in space and time may also be related to predator–prey relationships. Notably, the two large sharks, in particular the *C*. *taurus*, are opportunistic feeders consuming a wide variety of fish (teleosts and elasmobranchs) and are likely predators to the two mesopredatory teleost species, especially *A*. *japonicus* (Smale, 2005). Dynamics between predators and prey are often complex, but in theory, predators should attempt to match the distribution of their prey, and prey should avoid areas of high predation risk (Barnett & Semmens, 2012). Therefore, although there is some overlap in broad‐scale habitat use between the sharks and teleosts in Algoa Bay, the latter might be avoiding the former, resulting in the significant differences in fine‐scale habitat use detected.

## 5 CONCLUSION

This study emphasizes the importance of investigating multiple species when assessing the relative degree to which a specific environment, that is, Algoa Bay, is used, which has important implications when considering the development or adjustment of existing marine spatial management measures. Our findings demonstrate that Algoa Bay appears to be an important environment for all the tagged predators, and that use of the bay and movement patterns in the bay are variable and species specific. This sheltered coastal bay with distinct seasonal oceanographic characteristics provides not only an abundance of food but also protection from strong water currents and wave action. Furthermore, the presence of islands, particularly Bird Island with a seal colony, provides foraging opportunities for *C*. *carcharias*, and reefs in the western portion of the bay provide suitable habitat for *C*. *taurus*, whereas the Sundays and Swartkops estuaries and adjacent nearshore areas provide suitable nursery and foraging habitats for *A*. *japonicus* and *L*. *amia*. Consequently, the study species, and possibly others, are likely afforded some protection while in the no‐take zones of the current GAENP MPA, but they are vulnerable when leaving the bay and using other habitats, particularly the estuaries, where many of the teleosts were recaptured by recreational anglers subsequent to tagging.

## AUTHOR CONTRIBUTIONS

Data generation, data analysis, manuscript preparation, and editing: Taryn S. Murray and Chantel Elston. Study conceptualization, funding recipient that allowed for this work to be conducted, manuscript editing, and proofing: Malcolm J. Smale. Manuscript editing and proofing: Amber‐Robyn Childs. Manuscript editing and proofing: Matthew L. Dicken. Study conceptualization and funding: Paul D. Cowley.

## FUNDING INFORMATION

African Coelacanth Ecosystem Programme; Bayworld; National Research Foundation; Ocean Tracking Network; Save Our Seas Foundation; Shallow Marine and Coastal Research Infrastructure Programme; South African Institute for Aquatic Biodiversity.

## Supporting information


**Table S1.** Summary of the monitoring information for juvenile (J), subadult (SA), and adult (A) *Argyrosomus japonicus* (SA: 700–900 mm total length [TL]; A: > 900 mm TL), *Lichia amia* (SA: 400–700 mm fork length [FL]; A: > 700 mm FL), *Carcharias taurus* (J: <1.8 m TL; SA: ♂ 1.8–2.2 m TL, ♀ 1.8–2.4 m TL; A: ♂ >2.2 m TL, ♀ >2.4 m TL), and *Carcharodon carcharias* (J: 1.75–3.0 m TL; SA: ♂ 3.0–3.6 m TL, ♀ 3.0–4.8 m TL; A: ♂ >3.6 m TL, ♀ >4.8 m TL) tagged in Algoa Bay between August 2008 and July 2016. Fishes that were recaptured and killed prior to battery depletion are in boldface. *C. carcharias* tagged internally are denoted by gray cells.
