# Supplementary material for: Marine predators segregate interspecifically by space and time in a sheltered coastal bay
Source: J Fish Biol. 2024 May 9;106(5):1452–66. doi: 10.1111/jfb.15781 (PMC12120323; doi:10.1111/jfb.15781)
Supplement: Supplementary file 1 — Table S1. Summary of the monitoring information for juvenile (J), subadult (SA), and adult (A) Argyrosomus japonicus (SA: 700–900 mm total length [TL]; A: > 900 mm TL), Lichia amia (SA: 400–700 mm fork length [FL]; A: > 700 mm FL), Carcharias taurus (J: <1.8 m TL; SA: ♂ 1.8–2.2 m TL, ♀ 1.8–2.4 m TL; A: ♂ >2.2 m TL, ♀ >2.4 m TL), and Carcharodon carcharias (J: 1.75–3.0 m TL; SA: ♂ 3.0–3.6 m TL, ♀ 3.0–4.8 m TL; A: ♂ >3.6 m TL, ♀ >4.8 m TL) tagged in Algoa Bay between August 2008 and July 2016. Fishes that were recaptured and killed prior to battery depletion are in boldface. C. carcharias tagged internally are denoted by gray cells. [file JFB-106-1452-s001.docx]

**Marine predators segregate inter-specifically by space and time in a sheltered coastal bay**

Taryn S. Murray^1*^, Chantel Elston^1^, Malcolm J. Smale^2,3^, Amber-Robyn Childs^4^, Matthew L. Dicken^5,6^, Paul D. Cowley^1^

^1^ South African Institute for Aquatic Biodiversity, Private Bag X1015, Makhanda 6140, South Africa

^2^ Port Elizabeth Museum at Bayworld, P.O. Box 13147, Humewood, Gqebehra 6031, South Africa

^3^ Department of Zoology and Institute for Coastal and Marine Research, Nelson Mandela University, P.O. Box 77000, Gqeberha 6031, South Africa

^4^ Department of Ichthyology and Fisheries Science, Rhodes University, Makhanda, South Africa

^5^ KwaZulu-Natal Sharks Board, Umhlanga Rocks, South Africa

^6^ Institute for Coastal and Marine Research, Oceans Sciences Campus, Nelson Mandela University, P.O. Box 77000, Gqeberha 6031, South Africa

*corresponding author: TS.Murray@saiab.nrf.ac.za

**Table S1**: Summary of the monitoring information for juvenile (J), sub-adult (SA) and adult (A) *Argyrosomus japonicus* (SA: 700–900 mm total length [TL]; A: > 900 mm TL), *Lichia amia* (S-A: 400–700 mm fork length [FL]; A: > 700 mm FL), *Carcharias taurus* (J: <1.8 m TL; SA: ♂ 1.8 – 2.2 m TL, ♀ 1.8 – 2.4 m TL; A: ♂ >2.2 m TL, ♀ >2.4 m TL) and *Carcharodon carcharias* (J: 1.75 – 3.0 m TL; S-A: ♂ 3.0 – 3.6 m TL, ♀ 3.0 – 4.8 m TL; A: ♂ >3.6 m TL, ♀ > 4.8 m TL) tagged in Algoa Bay between August 2008 and July 2016. Fishes that were recaptured and killed prior to battery depletion are in boldface. *Carcharodon carcharias* tagged internally are denoted by grey cells.

| **Tag ID** | **Length**  **(mm TL, mm FL)** | **Life stage** | **Sex (sharks only)** | **Date tagged** | **No. detections** | **Days monitored** | **Days detected** | **Overall detection index (DI_total_)** | **Tag type** | **Estimated battery life (days)** |
| --- | --- | --- | --- | --- | --- | --- | --- | --- | --- | --- |
| *Argyrosomus japonicus* | |  |  |  |  |  |  |  |  |  |
| A69-9001-23700 | 740 mm TL | Sub-adult | - | 21-Apr-15 | 6 | 318 | 4 | 0.013 | V16-4L, 45 s | 3197 |
| A69-9001-23699 | 740 mm TL | Sub-adult | - | 27-Aug-15 | 36 | 1954 | 5 | 0.003 | V16-4L, 45 s | 3197 |
| A69-9001-23683 | 755 mm TL | Sub-adult | - | 04-Aug-15 | 136 | 1232 | 23 | 0.019 | V16-4L, 45 s | 3197 |
| **A69-9001-23684a** | **795 mm TL** | **Sub-adult** | **-** | **04-Aug-15** | **8** | **71** | **1** | **0.014** | **V16-4L, 45 s** | **59** |
| **A69-9001-23698a** | **795 mm TL** | **Sub-adult** | **-** | **27-Aug-15** | **11** | **48** | **1** | **0.021** | **V16-4L, 45 s** | **36** |
| A69-9001-23708 | 800 mm TL | Sub-adult | - | 23-Sep-15 | NA | NA | NA | NA | V16-4L, 45 s | 3197 |
| A69-9001-23706 | 805 mm TL | Sub-adult | - | 21-Apr-15 | 196 | 1682 | 9 | 0.005 | V16-4L, 45 s | 3197 |
| **A69-9001-24442a** | **850 mm TL** | **Sub-adult** | **-** | **23-Aug-14** | **NA** | **NA** | **NA** | **NA** | **V16-4L, 45 s** | **64** |
| A69-9001-24445 | 880 mm TL | Sub-adult | - | 24-Aug-14 | 131 | 2322 | 13 | 0.006 | V16-4L, 45 s | 3197 |
| **A69-9001-23741a** | **880 mm TL** | **Sub-adult** | **-** | **17-Sep-15** | **NA** | **NA** | **NA** | **NA** | **V16-4L, 45 s** | **0** |
| A69-9001-24438 | 910 mm TL | Adult | - | 24-Aug-14 | NA | NA | NA | NA | V16-4L, 45 s | 3197 |
| **A69-9001-23682a** | **970 mm TL** | **Adult** | **-** | **04-Aug-15** | **NA** | **NA** | **NA** | **NA** | **V16-4L, 45 s** | **0** |
| A69-9001-23679 | 980 mm TL | Adult | - | 19-Jul-15 | 68 | 253 | 10 | 0.040 | V16-4L, 45 s | 3197 |
| A69-9001-24443 | 1010 mm TL | Adult | - | 23-Aug-14 | 133 | 2323 | 4 | 0.002 | V16-4L, 45 s | 3197 |
| A69-9001-24444a | 1015 mm TL | Adult | - | 23-Aug-14 | 2 | 39 | 1 | 0.026 | V16-4L, 45 s | 3197 |
| A69-9001-23707 | 1020 mm TL | Adult | - | 23-Sep-15 | 74 | 653 | 8 | 0.012 | V16-4L, 45 s | 3197 |
| A69-1303-50207 | 1110 mm TL | Adult | - | 30-Aug-08 | 4376 | 1812 | 504 | 0.278 | V16-4L, 90s | 1160 |
| A69-1303-65066 | 1130 mm TL | Adult | - | 04-Sep-10 | 3117 | 1030 | 169 | 0.164 | V16-4L, 90s | 1160 |
| A69-9001-23742 | 1150 mm TL | Adult | - | 03-Oct-15 | 13607 | 1917 | 474 | 0.203 | V16-4L, 45 s | 3197 |
| A69-1303-65064 | 1160 mm TL | Adult | - | 04-Sep-10 | 9479 | 868 | 238 | 0.274 | V16-4L, 90s | 1160 |
| A69-1303-65063 | 1175 mm TL | Adult | - | 04-Sep-10 | 16243 | 1033 | 358 | 0.347 | V16-4L, 90s | 1160 |
| A69-9001-24435 | 1180 mm TL | Adult | - | 18-Oct-14 | 3158 | 2267 | 71 | 0.025 | V16-4L, 45 s | 3197 |
| A69-9001-24439 | 1260 mm TL | Adult | - | 24-Aug-14 | 11751 | 2322 | 266 | 0.115 | V16-4L, 45 s | 3197 |
| A69-1303-65065 | 1280 mm TL | Adult | - | 04-Sep-10 | 6023 | 1034 | 212 | 0.205 | V16-4L, 90s | 1160 |
| A69-9001-24437 | 1490 mm TL | Adult | - | NA | NA | NA | NA | NA | V16-4L, 45 s | 3197 |
| *Lichia amia* |  |  |  |  |  |  |  |  |  |  |
| A69-1105-217 | 400 mm FL | Sub-adult | - | 18-Oct-11 | 27 | 3363 | 9 | 0.003 | V13 D |  |
| A69-1303-10888 | 439 mm FL | Sub-adult | - | 28-Aug-13 | 7 | 80 | 2 | 0.025 | V13-1L, 40 s | 436 |
| A69-1303-10887 | 502 mm FL | Sub-adult | - | 28-Jul-13 | 220 | 173 | 7 | 0.040 | V13-1L, 40 s | 436 |
| A69-9001-23676 | 600 mm FL | Sub-adult | - | 10-Jul-16 | 752 | 873 | 21 | 0.024 | V16-4L, 45 s | 3197 |
| **A69-1303-50369a** | **613 mm FL** | **Sub-adult** | **-** | **25-Jan-13** | **153** | **51** | **19** | **0.373** | **V16-4H, 120 s** | **51** |
| **A69-1303-32996a** | **640 mm FL** | **Sub-adult** | **-** | **19-Aug-14** | **51** | **68** | **8** | **0.118** | **V16-4L, 60 s** | **75** |
| A69-9001-24431a | 670 mm FL | Sub-adult | - | 02-Oct-14 | 3657 | 140 | 57 | 0.407 | V16-4L, 45 s | 3197 |
| A69-1303-50376a | 675 mm FL | Sub-adult | - | 01-Nov-14 | 7110 | 384 | 69 | 0.180 | V16-4H, 120 s | 1625 |
| **A69-1303-50377a** | **675 mm FL** | **Sub-adult** | **-** | **03-Nov-14** | **770** | **257** | **29** | **0.113** | **V16-4H, 120 s** | **269** |
| A69-9001-24432 | 680 mm FL | Sub-adult | - | 02-Oct-14 | 134 | 1179 | 17 | 0.014 | V16-4L, 45 s | 3197 |
| A69-9001-25860 | 690 mm FL | Sub-adult | - | 19-Dec-14 | 133 | 10 | 6 | 0.600 | V16-4H, 90 s | 1018 |
| **A69-9001-24433a** | **695 mm FL** | **Sub-adult** | **-** | **17-Oct-14** | **1712** | **225** | **69** | **0.307** | **V16-4L, 45 s** | **226** |
| A69-9001-24434 | 698 mm FL | Sub-adult | - | 17-Oct-14 | 59 | 196 | 6 | 0.031 | V16-4L, 45 s | 3197 |
| A69-1303-50370 | 724 mm FL | Adult | - | 25-Jan-13 | 869 | 30 | 14 | 0.467 | V16-4H, 120 s | 1635 |
| **A69-1303-32996b** | **730 mm FL** | **Adult** | **-** | **23-Jan-15** | **3028** | **107** | **22** | **0.206** | **V16-4L, 45 s** | **120** |
| **A69-9001-23675a** | **745 mm FL** | **Adult** | **-** | **15-Feb-15** | **2882** | **552** | **42** | **0.076** | **V16-4L, 45 s** | **556** |
| A69-1303-50375 | 750 mm FL | Adult | - | 01-Nov-14 | 1456 | 304 | 84 | 0.276 | V16-4H, 120 s | 1625 |
| **A69-9001-24430b** | **750 mm FL** | **Adult** | **-** | **18-Dec-14** | **2247** | **849** | **28** | **0.033** | **V16-4L, 45 s** | **32** |
| A69-9001-25859 | 750 mm FL | Adult | - | 18-Dec-14 | 236 | 686 | 30 | 0.044 | V16-4H, 90 s | 1018 |
| A69-9001-24430c | 755 mm FL | Adult | - | 23-Jan-15 | 3257 | 2170 | 125 | 0.058 | V16-4L, 45 s | 3165 |
| A69-1303-50378 | 815 mm FL | Adult | - | 02-Aug-12 | 630 | 418 | 23 | 0.055 | V16-4H, 120 s | 1635 |
| A69-9001-23740 | 830 mm FL | Adult | - | 23-Aug-15 | NA | NA | NA | NA | V16-4L, 45 s | 3197 |
| A69-1303-50381 | 840 mm FL | Adult | - | 02-Aug-12 | 2178 | 1579 | 51 | 0.032 | V16-4H, 120 s | 1635 |
| *Carcharias taurus* |  |  |  |  |  |  |  |  |  |  |
| A69-1303-33012 | 1.33 m TL | Juvenile | Female | 05-May-13 | 622 | 78 | 37 | 0.474 | V16-6L, 120 s | 3285 |
| A69-1303-32505 | 1.35 m TL | Juvenile | Male | 14-Oct-15 | 34313 | 1526 | 698 | 0.457 | V16-6x, 120 s | 2930 |
| A69-1303-32510 | 1.42 m TL | Juvenile | Male | 14-Oct-15 | 48783 | 1906 | 725 | 0.380 | V16-6x, 120 s | 2930 |
| A69-1303-32509 | 1.48 m TL | Juvenile | Male | 14-Oct-15 | 13085 | 1485 | 442 | 0.298 | V16-6x, 120 s | 2930 |
| A69-1303-32507 | 1.5 m TL | Juvenile | Male | 14-Oct-15 | 16628 | 1730 | 489 | 0.283 | V16-6x, 120 s | 2930 |
| A69-1303-32162 | 1.51 m TL | Juvenile | Male | 12-Jan-15 | 363 | 195 | 31 | 0.159 | V16-6x, 120 s | 2870 |
| A69-1303-33020 | 1.52 m TL | Juvenile | Female | 25-Sep-14 | 52130 | 2290 | 656 | 0.286 | V16-6L, 120 s | 3285 |
| A69-1303-32503 | 1.59 m TL | Juvenile | Female | 12-Jan-15 | 34635 | 2181 | 775 | 0.355 | V16-6x, 120 s | 2930 |
| A69-1303-33024 | 1.62 m TL | Juvenile | Male | 19-Nov-14 | 92 | 6 | 3 | 0.500 | V16-6L, 120 s | 3285 |
| A69-1303-32508 | 1.62 m TL | Juvenile | Male | 14-Oct-15 | 7467 | 1906 | 215 | 0.113 | V16-6x, 120 s | 2930 |
| A69-1303-32624 | 1.65 m TL | Juvenile | Female | 16-Dec-14 | 27424 | 2208 | 759 | 0.344 | V16-6L, 120 s | 3102 |
| A69-1303-9420 | 1.69 m TL | Juvenile | Male | 07-Feb-13 | 6499 | 1250 | 216 | 0.173 | V16-6x, 165 s | 3650 |
| A69-1303-9418 | 1.74 m TL | Juvenile | Female | 02-Feb-13 | 2318 | 443 | 108 | 0.244 | V16-6x, 165 s | 3650 |
| A69-1303-32511 | 1.76 m TL | Juvenile | Female | 14-Oct-15 | 69701 | 1906 | 753 | 0.395 | V16-6x, 120 s | 2930 |
| A69-1303-9416 | 1.80 m TL | Sub-adult | Female | 12-Jan-13 | 2912 | 737 | 74 | 0.100 | V16-6x, 165 s | 3650 |
| A69-1303-9415 | 1.82 m TL | Sub-adult | Female | 12-Jan-13 | 17512 | 754 | 261 | 0.346 | V16-6x, 165 s | 3650 |
| A69-1303-32625 | 1.83 m TL | Sub-adult | Male | 16-Dec-14 | 2670 | 356 | 62 | 0.174 | V16-6L, 120 s | 3102 |
| A69-1303-32506 | 1.85 m TL | Sub-adult | Male | 14-Oct-15 | 17783 | 1906 | 392 | 0.206 | V16-6x, 120 s | 2930 |
| A69-1303-33007 | 1.86 m TL | Sub-adult | Female | 07-Feb-13 | 954 | 221 | 56 | 0.253 | V16-6x, 120 s | 3285 |
| A69-1303-33022 | 1.86 m TL | Sub-adult | Male | 25-Sep-14 | 42989 | 2290 | 407 | 0.178 | V16-6L, 120 s | 3285 |
| A69-1303-33010 | 1.88 m TL | Sub-adult | Female | 21-Feb-13 | 10381 | 2871 | 186 | 0.065 | V16-6L, 120 s | 3285 |
| A69-1303-33011 | 1.88 m TL | Sub-adult | Female | 21-Feb-13 | 22480 | 2871 | 438 | 0.153 | V16-6L, 120 s | 3285 |
| A69-1303-9421 | 1.89 m TL | Sub-adult | Male | 07-Feb-13 | 3015 | 737 | 180 | 0.244 | V16-6x, 165 s | 3650 |
| A69-1303-33008 | 1.89 m TL | Sub-adult | Female | 21-Feb-13 | 14 | 2 | 1 | 0.500 | V16-6L, 120 s | 3285 |
| A69-1303-33021 | 1.89 m TL | Sub-adult | Male | 25-Sep-14 | 14382 | 2290 | 422 | 0.184 | V16-6L, 120 s | 3285 |
| A69-1303-32623 | 1.89 m TL | Sub-adult | Female | 16-Dec-14 | 17787 | 2208 | 384 | 0.174 | V16-6L, 120 s | 3102 |
| A69-1303-33023 | 1.94 m TL | Sub-adult | Female | 19-Nov-14 | 12631 | 2235 | 333 | 0.149 | V16-6L, 120 s | 3285 |
| A69-1303-32502 | 1.97 m TL | Sub-adult | Male | 12-Jan-15 | 7347 | 2181 | 222 | 0.102 | V16-6x, 120 s | 2930 |
| A69-1303-33009 | 1.98 m TL | Sub-adult | Male | 21-Feb-13 | 12223 | 2871 | 419 | 0.146 | V16-6L, 120 s | 3285 |
| A69-1303-32504 | 2.00 m TL | Sub-adult | Female | 12-Jan-15 | 1690 | 2181 | 107 | 0.049 | V16-6x, 120 s | 2930 |
| A69-1303-33026 | 2.03 m TL | Sub-adult | Female | 16-Dec-14 | 10598 | 2208 | 287 | 0.130 | V16-6L, 120 s | 3285 |
| A69-1303-9419 | 2.10 m TL | Sub-adult | Male | 02-Feb-13 | 9637 | 689 | 111 | 0.161 | V16-6x, 165 s | 3650 |
| A69-1303-33014 | 2.12 m TL | Sub-adult | Female | 13-May-13 | 165809 | 2790 | 1054 | 0.378 | V16-6L, 120 s | 3285 |
| A69-1303-8699 | 2.13 m TL | Sub-adult | Male | 07-Feb-13 | 1513 | 1125 | 191 | 0.170 | V16-6x, 270 s | 3650 |
| A69-1303-32626 | 2.15 m TL | Sub-adult | Female | 19-Dec-14 | 45826 | 2205 | 417 | 0.189 | V16-6L, 120 s | 3102 |
| A69-1303-33025 | 2.18 m TL | Sub-adult | Female | 16-Dec-14 | 62587 | 2208 | 709 | 0.321 | V16-6L, 120 s | 3285 |
| A69-1303-33015 | 2.23 m TL | Sub-adult | Female | 13-May-13 | 96320 | 2790 | 590 | 0.211 | V16-6L, 120 s | 3285 |
| A69-1303-33019 | 2.24 m TL | Sub-adult | Female | 05-Nov-13 | 15502 | 2614 | 174 | 0.067 | V16-6L, 120 s | 3285 |
| A69-1303-33017 | 2.32 m TL | Sub-adult | Female | 09-Oct-13 | 54324 | 2641 | 400 | 0.151 | V16-6L, 120 s | 3285 |
| A69-1303-9417 | 2.33 m TL | Sub-adult | Male | 23-Jan-13 | 5783 | 719 | 130 | 0.181 | V16-6x, 165 s | 3650 |
| A69-1303-32627 | 2.35 m TL | Sub-adult | Female | 19-Dec-14 | 59648 | 2205 | 491 | 0.223 | V16-6L, 120 s | 3102 |
| A69-1303-33013 | 2.40 m TL | Adult | Female | 13-May-13 | 118104 | 1349 | 612 | 0.454 | V16-6L, 120 s | 3285 |
| A69-1303-55864 | 2.45 m TL | Adult | Male | 26-Jul-11 | NA | NA | NA | NA | V16-5x | 899 |
| A69-1303-33016 | 2.52 m TL | Adult | Female | 09-Oct-13 | 4668 | 402 | 79 | 0.197 | V16-6L, 120 s | 3285 |
| A69-1303-55865 | 2.60 m TL | Adult | Female | 19-Nov-12 | 1111 | 1013 | 87 | 0.086 | V16-5x | 899 |
| A69-1303-33018 | 2.78 m TL | Adult | Female | 05-Nov-13 | 8120 | 2614 | 235 | 0.090 | V16-6L, 120 s | 3285 |
| A69-1303-55863 | 2.87 m TL | Adult | Female | 26-Jul-11 | 289 | 315 | 13 | 0.041 | V16-5x | 899 |
| A69-1303-32161 | 3.00 m TL | Adult | Female | 19-Dec-14 | 44914 | 2205 | 407 | 0.185 | V16-6x, 120 s | 2870 |
| *Carcharodon carcharias* | |  |  |  |  |  |  |  |  |  |
| A69-1303-34162 | 1.80 m TL | Juvenile | Unknown | 05-Nov-12 | 2714 | 578 | 192 | 0.332 | V16-5H | 1544 |
| A69-1303-33041 | 2.00 m TL | Juvenile | Female | 20-May-13 | 11150 | 750 | 389 | 0.224 | V16-5H, 120 s | 899 |
| A69-1303-34161 | 2.00 m TL | Juvenile | Female | 30-Sep-12 | 3193 | 549 | 290 | 0.528 | V16-5H | 1544 |
| A69-1303-52619 | 2.00 m TL | Juvenile | Male | 19-Nov-12 | 1026 | 604 | 151 | 0.250 | V16-6L | 3650 |
| **A69-1303-33043** | **2.20 m TL** | **Juvenile** | **Male** | **20-May-13** | 2040 | **388** | **67** | **0.173** | **V16-5H, 120s** | **745** |
| A69-1303-33046 | 2.20 m TL | Juvenile | Male | 28-Jun-13 | 2387 | 695 | 127 | 0.183 | V16-5H, 120 s | 899 |
| A69-1303-8697 | 2.20 m TL | Juvenile | Female | 10-Nov-12 | 36 | 16 | 8 | 0.500 | V16-6H | 3650 |
| A69-1303-52618 | 2.30 m TL | Juvenile | Female | 19-Nov-12 | 294 | 1455 | 47 | 0.032 | V16-6L | 3650 |
| A69-1303-8698 | 2.42 m TL | Juvenile | Female | 10-Nov-12 | 275 | 177 | 75 | 0.424 | V16-6H | 3650 |
| A69-1303-33049 | 2.50 m TL | Juvenile | Male | 28-Jun-13 | 357 | 12 | 10 | 0.833 | V16-5H, 120 s | 899 |
| A69-1303-33050 | 2.50 m TL | Juvenile | Female | 26-Jul-13 | 4705 | 344 | 124 | 0.360 | V16-5H, 120 s | 899 |
| A69-1303-34165 | 2.50 m TL | Juvenile | Female | 20-May-13 | 1002 | 291 | 78 | 0.268 | V16-5H | 1544 |
| A69-1303-44481 | 2.50 m TL | Juvenile | Unknown | 22-Jun-12 | 342 | 342 | 22 | 0.064 | V16-5H | 1544 |
| A69-1303-32630 | 2.80 m TL | Juvenile | Female | 29-Jul-14 | 7110 | 413 | 261 | 0.632 | V16-5H, 120 s | 899 |
| A69-1303-34163 | 2.80 m TL | Juvenile | Female | 20-May-13 | 2713 | 176 | 143 | 0.813 | V16-5H | 1544 |
| A69-1303-34166 | 2.80 m TL | Juvenile | Male | 19-Nov-12 | 12 | 324 | 2 | 0.006 | V16-5H | 1544 |
| A69-1303-32629 | 3.00 m TL | Juvenile | Female | 29-Jul-14 | 1541 | 82 | 78 | 0.090 | V16-5H, 120 s | 899 |
| A69-1303-33040 | 3.00 m TL | Juvenile | Female | 20-May-13 | 5725 | 241 | 170 | 0.705 | V16-5H, 120 s | 899 |
| A69-1303-33042 | 3.00 m TL | Juvenile | Female | 20-May-13 | 665 | 184 | 46 | 0.250 | V16-5H, 120 s | 899 |
| A69-1303-33044 | 3.00 m TL | Juvenile | Female | 20-May-13 | 8209 | 545 | 265 | 0.486 | V16-5H, 120 s | 899 |
| A69-1303-34164 | 3.00 m TL | Juvenile | Female | 10-Nov-12 | 10 | 1326 | 2 | 0.002 | V16-5H | 1544 |
| A69-1303-34170 | 3.00 m TL | Juvenile | Female | 23-Nov-12 | 374 | 149 | 46 | 0.309 | V16-5H | 1544 |
| A69-1303-34171 | 3.00 m TL | Juvenile | Female | 23-Nov-12 | 13 | 48 | 4 | 0.083 | V16-5H | 1544 |
| A69-1303-44477 | 3.00 m TL | Juvenile | Male | 22-Jun-12 | 485 | 130 | 62 | 0.477 | V16-5H | 1544 |
| A69-1303-44478 | 3.00 m TL | Juvenile | Female | 22-Jun-12 | 5897 | 617 | 368 | 0.596 | V16-5H | 1544 |
| A69-1303-44480 | 3.00 m TL | Juvenile | Unknown | 22-Jun-12 | 1166 | 120 | 97 | 0.808 | V16-5H | 1544 |
| A69-1303-44482 | 3.00 m TL | Juvenile | Male | 24-Jul-12 | 289 | 485 | 29 | 0.060 | V16-5H | 1544 |
| A69-1303-33045 | 3.20 m TL | Sub-adult | Male | 28-Jun-13 | 6051 | 820 | 215 | 0.262 | V16-5H, 120 s | 899 |
| A69-1303-33047 | 3.20 m TL | Sub-adult | Female | 28-Jun-13 | 3264 | 915 | 62 | 0.068 | V16-5H, 120 s | 899 |
| A69-1303-34168 | 3.20 m TL | Sub-adult | Male | 19-Nov-12 | 150 | 317 | 13 | 0.041 | V16-5H | 1544 |
| A69-1303-33048 | 3.50 m TL | Sub-adult | Male | 28-Jun-13 | 3241 | 495 | 205 | 0.414 | V16-5H, 120 s | 899 |
| A69-1303-34169 | 3.50 m TL | Sub-adult | Female | 23-Nov-12 | 36 | 80 | 8 | 0.100 | V16-5H | 1544 |
| A69-1303-34172 | 3.50 m TL | Sub-adult | Female | 23-Nov-12 | 477 | 551 | 50 | 0.091 | V16-5H | 1544 |
| A69-1303-44476 | 3.50 m TL | Sub-adult | Female | 22-Jun-12 | 2132 | 142 | 121 | 0.852 | V16-5H | 1544 |
| A69-1303-44479 | 3.50 m TL | Sub-adult | Unknown | 22-Jun-12 | 544 | 31 | 26 | 0.839 | V16-5H | 1544 |
| A69-1303-32631 | 5.20 m TL | Adult | Female | 29-Jul-14 | 1 | 1230 | 1 | 0.001 | V16-5H, 120 s | 899 |
| A69-1303-46787 | Unknown | Unknown | Female | 22-Jun-12 | 1751 | 142 | 99 | 0.697 | V16-5H | 1544 |
